# Comparison of Simoa^TM^ and Ella^TM^ to assess serum neurofilament‐light chain in multiple sclerosis

**DOI:** 10.1002/acn3.51355

**Published:** 2021-04-08

**Authors:** Audrey Gauthier, Sébastien Viel, Magali Perret, Guillaume Brocard, Romain Casey, Christine Lombard, Sabine Laurent‐Chabalier, Marc Debouverie, Gilles Edan, Sandra Vukusic, Christine Lebrun‐Frénay, Jérôme De Sèze, David Axel Laplaud, Giovanni Castelnovo, Olivier Gout, Aurélie Ruet, Thibault Moreau, Olivier Casez, Pierre Clavelou, Eric Berger, Hélène Zephir, Sophie Trouillet‐Assant, Eric Thouvenot, OFSEP Investigators

**Affiliations:** ^1^ Paris Sciences et Lettres ‐ École Pratique des Hautes Études Paris France; ^2^ Immunology laboratory Hospices Civils de Lyon Lyon Sud Hospital Pierre‐Bénite France; ^3^ International Center of Research in Infectiology Lyon University INSERM U1111 CNRS UMR 5308 ENS UCBL Lyon France; ^4^ Université de Lyon Université Claude Bernard Lyon 1 Lyon F‐69000 France; ^5^ Hospices Civils de Lyon Service de Neurologie, sclérose en plaques, pathologies de la myéline et neuro‐inflammation Bron F‐69677 France; ^6^ Observatoire Français de la Sclérose en Plaques Centre de Recherche en Neurosciences de Lyon INSERM 1028 et CNRS UMR 5292 Lyon F‐69003 France; ^7^ EUGENE DEVIC EDMUS Foundation against multiple sclerosis, state‐approved foundation Bron F‐69677 France; ^8^ Department of Biostatistics Clinical Epidemiology, Public Health, and Innovation in Methodology CHU Nîmes Univ. Montpellier Nîmes France; ^9^ Department of Neurology Nancy University Hospital Nancy France; ^10^ Université de Lorraine APEMAC Nancy F‐54000 France; ^11^ CHU Pontchaillou Rennes CIC1414 INSERM, F‐35000 France; ^12^ Department of Neurology UR2CA URRIS CRCSEP Centre Hospitalier Universitaire Pasteur2 Université Nice Côte d'Azur Nice France; ^13^ Department of Neurology and Clinical Investigation Center CIC 1434, INSERM 1434 CHU de Strasbourg Strasbourg F‐67000 France; ^14^ Department of Neurology and CIC015 INSERM CHU de Nantes Nantes F‐44093 France; ^15^ CRTI‐Inserm U1064 Nantes F‐44000 France; ^16^ Department of Neurology CHU Nîmes Univ Montpellier Nîmes France; ^17^ Department of Neurology Fondation Rothschild Paris F‐75000 France; ^18^ Univ. Bordeaux Bordeaux F‐33000 France; ^19^ INSERM U1215 Neurocentre Magendie Bordeaux F‐33000 France; ^20^ Department of Neurology CHU de Bordeaux CIC Bordeaux CIC1401 Bordeaux F‐33000 France; ^21^ Department of Neurology CHU de Dijon EA4184 Dijon F‐21000 France; ^22^ Department of Neurology CHU Grenoble Alpes La Tronche/Grenoble F‐38700 France; ^23^ Department of Neurology CHU Clermont‐Ferrand Clermont‐Ferrand F‐63000 France; ^24^ Université Clermont Auvergne Inserm, Neuro‐Dol Clermont‐Ferrand F‐63000 France; ^25^ Department of Neurology CHU Besançon Besançon F‐25030 France; ^26^ CHU Lille CRCSEP Lille Univ Lille Lille U1172, F‐59000 France; ^27^ Lyon Sud Hospital Pierre‐Bénite France; ^28^ Institut de Génomique Fonctionnelle Univ. Montpellier CNRS INSERM Montpellier Cedex 5 F‐34094 France

## Abstract

We compared Simoa^TM^ and Ella^TM^ immunoassays to assess serum neurofilament‐light chain levels in 203 multiple sclerosis patients from the OFSEP HD study. There was a strong correlation (ρ = 0.86, *p* < 0.0001) between both platforms. The Ella^TM^ instrument overestimated values by 17%, but as the data were linear (*p* = 0.57), it was possible to apply a correction factor to Ella^TM^ results. As for Simoa^TM^, serum neurofilament‐light chain levels measured by Ella^TM^ were correlated with age and EDSS and were significantly higher in active multiple sclerosis, suggesting that these assays are equivalent and can be used in routine clinical practice.

## Introduction

Neurofilaments (Nf) are major components of the neuronal cytoskeleton, consisting predominantly of three subunits: Nf‐light (NfL), Nf‐medium and Nf‐heavy chains.^1^ Upon neuro‐axonal damage of the central nervous system (CNS), NfL is released into the extracellular space and is detectable in the cerebrospinal fluid and blood.^2^ Thus, NfL levels are increased proportionally to the degree of damage,^2^ making serum NfL levels a useful biomarker for diagnosing and predicting disease progression of a variety of CNS disorders, including multiple sclerosis (MS).^3^ In MS, serum NfL is correlated with several factors including age, Expanded Disability Status Scale (EDSS), disease activity and disease‐modifying treatments.^4^

Several ultrasensitive immunoassay technologies are available for quantification of serum NfL. The current reference method is the Single Molecular Array (Simoa™, Quanterix)^5^ using an antibody developed by Uman Diagnostics. Recently, several companies have acquired this antibody, allowing NfL quantification using the Simple Plex^TM^ Ella (Ella^TM^) microfluidic platform (ProteinSimple). The Ella^TM^ instrument allows rapid and ultra‐sensitive measurement of biomarkers.^6^ This platform allows quantitation of an analyte from 72 samples in a single disposable microfluidic cartridge, within 90 minutes (ProteinSimple, 2020). However, the comparability of the two technologies in measuring serum NfL levels in patients with MS remains to be determined.

The objective of this study was to compare the NfL values obtained using the Simoa^TM^ platform with Ella™ instrument in MS patients and healthy controls (HCs). Correlations of the serum NfL measures were performed to evaluate whether Ella^TM^ had good clinical performance in reflecting age, EDSS and disease activity, and could be routinely used to monitor MS patients in clinical practice.

## Materials and methods

### Serum samples

Anonymized serum samples were taken from 203 of the 1800 anticipated patients ≥15 years old with MS according to the revised McDonald diagnosis criteria included in the OFSEP "High Definition" cohort (NCT03981003), and from 30 HCs. Ethics approvals were obtained, and all patients and controls participated voluntarily in the study and provided written informed consent (Details in Supplementary materials and methods).

### Simoa^TM^ and Ella^TM^ NfL assay

Serum NfL concentrations were prospectively determined in parallel with the Simoa^TM^ Human Neurology 4‐Plex “A” kit (Quanterix Corp, Boston, MA) on Simoa^TM^ HD‐1 analyzer and Simple Plex^TM^ NfL Assay (ProteinSimple, CA, USA) on Ella^TM^ instrument, according to the manufacturers’ instructions. Ella™ was calibrated using the in‐cartridge factory standard curve and Simoa™ using the provided standards. All samples were measured in simplicate, on the same day, after a single thaw, with a 1:2 dilution for Ella^TM^ and 1:4 for Simoa^TM^. In each run, the HC, one control patient with active relapsing remitting MS (RRMS), and one high and one low concentration control sample provided with the kits were assayed. The lower limit of quantification is 0.241 pg/ml for Simoa^TM^ and 2.70 pg/ml for Ella^TM^.

### Statistical analysis

The intra‐assay coefficients of variation (CV) of manufacturer‐provided controls were automatically calculated in duplicate (Simoa^TM^) or internal triplicate (Ella^TM^). Repeatability tests were performed with samples at high (RRMS patient) and low (HC) concentrations by repeated measures for Simoa^TM^ (30 times each) and for Ella^TM^ (28 times and 25 times, respectively). Intra‐assay CV was calculated from the standard deviation of the average concentrations divided by the overall mean of the average concentrations.

Median NfL values obtained by each platform were compared using the Wilcoxon–Mann–Whitney test. Spearman correlation coefficients were calculated to assess the association between concentrations obtained by each platform, presented with 95% confidence interval (95%CI). The Bland–Altman method^7^ was used to measure mean difference and 95% limit of agreement between log‐transformed concentrations obtained by each platform. The regression relationship between the two platforms was evaluated using Passing–Bablok.^8^ Finally, correlations of serum NfL levels with clinical parameters were analyzed using linear regression (age, EDSS) or Wilcoxon–Mann–Whitney (e.g. RRMS *vs*. progressive MS).

Statistical analyses were performed on Prism 8.3.0.538 (GraphPad). A p‐value <0.05 was considered statistically significant.

### Data availability statement

Anonymized data will be shared by request from any qualified investigator.

## Results

**Figure 1:**
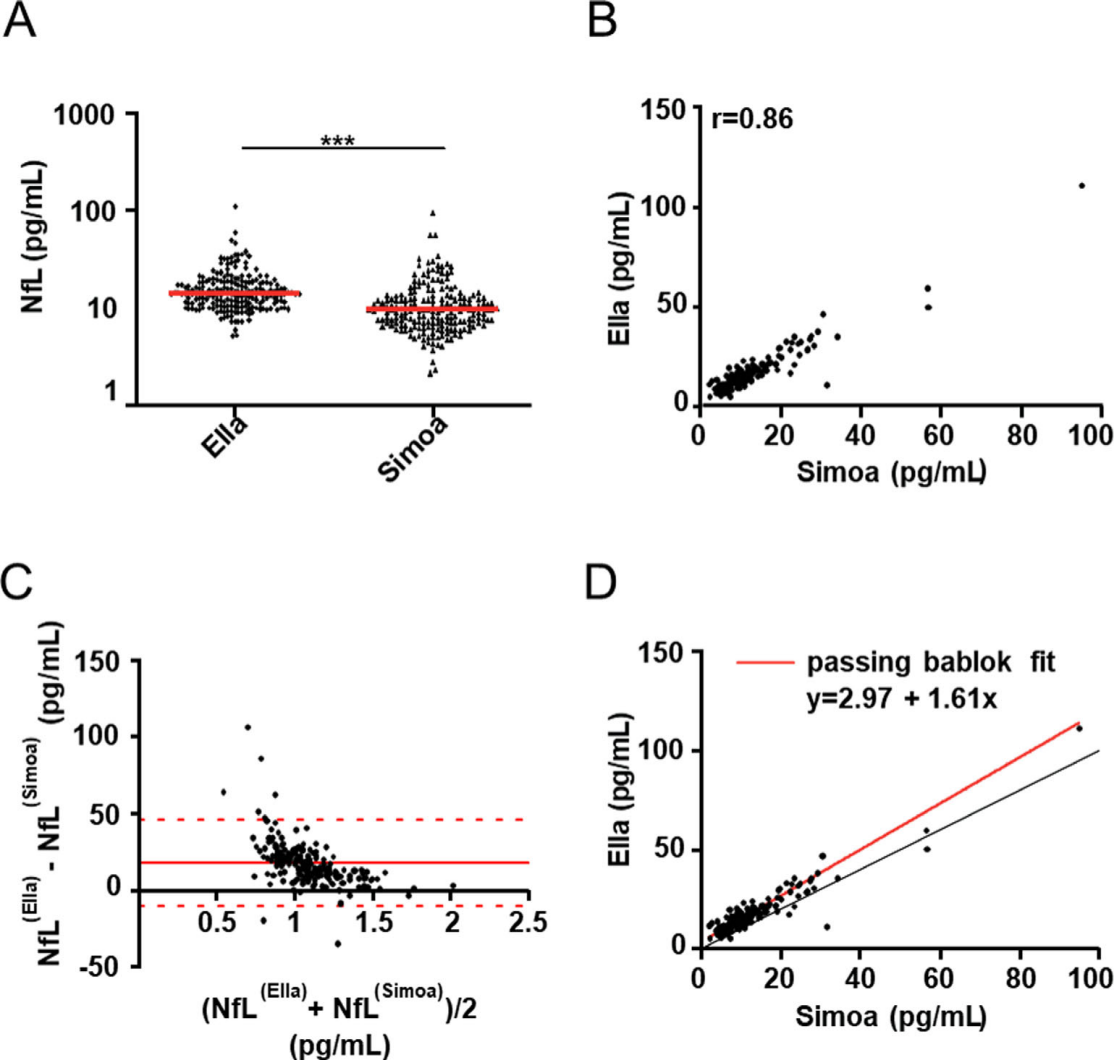
Properties of serum NfL values measured by the Simoa^TM^ and Ella^TM^ platforms. A, Quantitation of NfL concentration (pg/ml) in serum with Ella^TM^ and Simoa^TM^ platforms shown in logarithmic scale. Red lines represent median NfL level. The statistical difference was evaluated by Wilcoxon–Mann–Whitney with 203 samples. ***p < 0.001. B, Spearman correlation (r) between NfL concentration values obtained by the Ella^TM^ compared to the Simoa^TM^ instruments (*p* < 0001). C, Bland–Altman plots comparing agreement between NfL concentrations determined using the Simoa^TM^ and Ella^TM^ platforms. The solid red line represents the bias between assays (17.6%), the dashed red lines represent 95% limits of agreement (−10.61% to 45.81%). D, Passing–Bablok regression analysis of NfL concentration calculated on 203 samples by the Ella^TM^ compared to the Simoa^TM^ platform. It shows the value of slope (1.161) and intercept (2.917). Solid gray line: Passing–Bablok regression line; solid red line: identity line (x = y).

Repeatability tests were performed by measuring 25‐30 times one sample at low concentration (HC) and one sample at high concentration (RRMS patient) and showed similar CVs with both platforms (Supplementary Figure A). The mean [min‐max] intra‐assay CVs on Ella^TM^ technology was 2.12% [1.53‐2.70] vs 3.78% [2.93‐4.63] on Simoa^TM^ platform. The mean [min‐max] inter‐assay CV of the three runs was 12.93% [7.59‐18.27] on Ella^TM^ and 5.54% [5.08‐6.00] on Simoa^TM^. In MS patients, median serum NfL levels [interquartile range] measured by Ella^TM^ were higher than by Simoa^TM^ (13.90 pg/ml [10.73‐18.48] for Ella^TM^ *vs*. 9.46 pg/ml [6.94‐12.9] for Simoa^TM^, *p* < 0.001) (Figure 1A). Serum NfL levels were strongly correlated between the two technologies in MS patients (Spearman r = 0.86, 95% CI [0.821‐0.895]) (Figure 1B) and in HCs (Spearman r = 0.76, 95%CI [0.533‐0.882], Supplementary Figure B).

The Bland–Altman method depicted a mean bias of 17.6% for the NfL concentrations between the assays performed with the two technologies. Thus, Ella^TM^ showed a 17.6% “overestimation” compared with Simoa^TM^. Overall, 95% of observations were within the limit of agreement (Figure 1C). The slope of the Passing–Bablok regression line was 1.161 (95% CI [1.091‐1.240], *p* < 0.0001) and the intercept was 2.917 pg/ml (95% CI [2.132‐3.676], *p* < 0.0001). The 95% CI of intercept and slope values differ from zero and one, respectively, indicating a method agreement and allowing application of a correction coefficient.^9^ Moreover, the linearity test demonstrated no significant deviation from linearity between the two datasets (*p* = 0.57), suitable for concluding on method agreement (Figure 1D).

**Figure 2:**
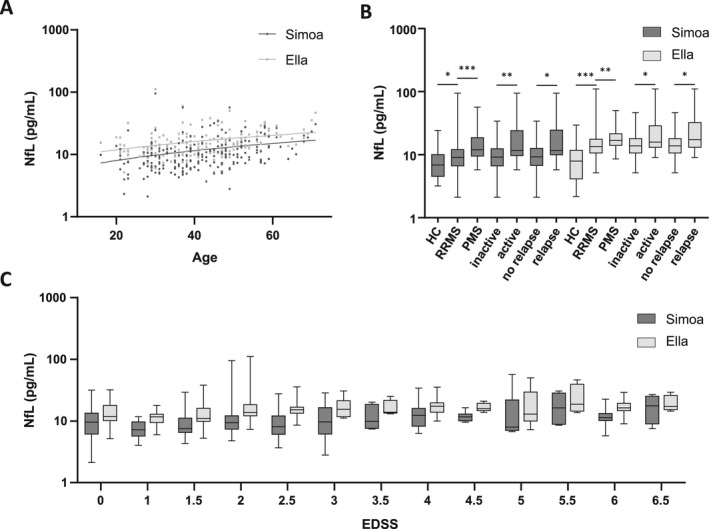
Comparison of serum NfL values measured by the Simoa^TM^ and Ella^TM^ platforms. A, Association of age with NfL concentration (pg/ml, shown in logarithmic scale) in serum determined by Ella^TM^ (light gray) and Simoa^TM^ (dark gray) platforms were estimated using the linear regression with 203 samples (b = 0.18, *p* = 0.002, r^2^ = 0.045 in Simoa^TM^ and b = 0.21, *p* < 001, r^2^ = 0.057 in Ella^TM^). B: Comparison of NfL levels (pg/ml, shown in logarithmic scale) in serum for HCs and MS patients, obtained by the Simoa^TM^ (dark gray, left) and the Ella^TM^ (light gray, right) instruments. Serum NfL levels were higher in RRMS patients than in HCs (*p* = 0.021 and *p* < 0001, respectively), higher in active MS than in inactive MS (*p* = 0.0080 and *p* = 0.0356, respectively), higher during relapses than in patients with a stable disease (*p* = 0.0153 and *p* = 0.0373, respectively), and lower in RRMS than in PMS patients (*p* = 0.0007 and *p* = 0.0021, respectively) (*p < 05, **p < 01, ***p < 001, ****p < 0001). C: Association of EDSS with NfL concentration (pg/ml, shown in logarithmic scale) in serum determined by Simoa^TM^ (left, dark gray boxplots) and Ella^TM^ (right, light gray boxplots) platforms were estimated using linear regression with 203 samples (b = 0.83, *p* = 0.026, r^2^ = 0.026 in Simoa^TM^ and b = 0.96, *p* = 0.015, r^2^ = 0.031 in Ella^TM^).

Both platforms exhibited significant correlations of serum NfL with age, EDSS and disease form (Figure 2). Especially, serum NfL levels were higher in RRMS patients than in age‐matched HCs, higher in active MS than in inactive MS, higher during relapses than in patients with a stable disease and higher in PMS than in RRMS patients with both platforms (Figure 2B). The last comparison was no longer significant in a multivariate model including age.

## Discussion

Blood NfL is a biomarker associated with several clinical parameters in MS.^10^ We showed that both Ella^TM^ and Simoa^TM^ platforms offer excellent sensitivity, detecting serum NfL concentrations in the picogram range, Simoa^TM^ platform offering the lowest inter‐assay imprecision at low analyte levels. A limitation of our study was restricting the analysis to three runs, making the inter‐assay CV harder to accurately define. The two systems use different methods to determine intra‐assay CV, using technical duplicate or triplicate readings, preventing direct comparison. However, Simple Plex^TM^ runs the samples in parallel at the same time, assuring the exact same conditions for replicate analysis, an advantage over the Simoa^TM^ platform that processes serial measures. Moreover, calibrators are directly integrated in the Simple Plex^TM^ cartridges, providing best calibration for each run.

The main finding of this study is the demonstration of a concordance between NfL levels measured using both platforms, even at low levels in the HC group. This is potentially the result of using the same anti‐NfL antibody and of heterophilic blockers limiting potential cross‐reaction between anti‐NfL antibody and antibodies in the serum for both platforms. However, we observed significant differences in absolute biomarker concentrations between these two instruments. Using different calibrators (naturally derived bovine NfL for Ella^TM^ and a recombinant human NfL for Simoa^TM^) has been associated with differences in NfL measure and could explain the differences in absolute values obtained by both assays.^2^ The NfL raw concentrations measured by Simoa^TM^ were globally lower vs Ella^TM^, as confirmed by the Bland–Altman plot. The “spike recovery” reported in the data sheet of the two assays is 68% for Simoa^TM^ NfL kit and 108% for Simple Plex^TM^ NfL, suggesting that Simoa^TM^ could underestimate the values of NfL by 17% compared to Ella^TM^ due to a greater effect of the serum matrix than in the Simple Plex^TM^ method. Passing–Bablok allowed the bias to be evaluated over the entire measurement range and the linear test shows that the data are linear (*p* = 0.57). Thus, it is possible to apply a correction factor 2.917. Therefore, Ella^TM^ technology, with the advantage of small footprint and a robust and cheaper platform, represents a reliable substitute for Simoa^TM^ to measure serum NfL.

Moreover, we demonstrate that serum NfL levels determined by Ella^TM^ show the same properties, concerning correlation of serum NfL with age, EDSS and disease activity. This is crucial, since future studies with Ella^TM^ can directly resume previous results already published using Simoa^TM^. However, NfL cannot be used in combination with other brain biomarkers that remain unavailable on this platform, such as glial fibrillary acidic protein, available on the Simoa^TM^ platform which currently has a larger range of biomarkers.

Although the Ella^TM^ platform showed a greater inter‐assay variation compared to Simoa^TM^, it seems an attractive choice for routine quantification of serum NfL considering the reduced cost, high performance and small footprint while maintaining a high concordance with Simoa^TM^. Serum NfL biomarker can be quantified using automated Ella^TM^ instrument to reliably and rapidly monitor disease activity and treatment in MS as well as in many other CNS pathological conditions, thus optimizing quality of care.

## Conflict of interest

*The authors declare that the research was conducted in the absence of any commercial or financial relationships that could be construed as a potential conflict of interest*. Audrey Gauthier: nothing to disclose; Sébastien Viel: nothing to disclose; Magali Perret: nothing to disclose; Sabine Laurent‐Chabalier: nothing to disclose; Marc Debouverie: nothing to disclose; Gilles Edan: consultancy and lecturing fees from Bayer‐Schering, Biogen, LFB, Merck, Novartis, Roche, Sanofi; research grants from Bayer, Biogen, Genzyme, Mercks, Novartis, Roche, Teva, and the ARSEP foundation. He has been principal investigator in phase 2 and 3 clinical studies conducted by Bayer, Biogen, Merck, Novartis, Sanofi‐Aventis Teva, and 4 academic programs (programmes hospitaliers de recherche clinique, PHRC) on MS sponsored by Rennes University Hospital; Sandra Vukusic: grants, personal fees and non‐financial support from Biogen, grants and personal fees from Geneuro, grants, personal fees and non‐financial support from Genzyme, grants and personal fees from Medday, grants, personal fees and non‐financial support from Merck‐Serono, grants, personal fees and non‐financial support from Novartis, grants, personal fees and non‐financial support from Roche, grants, personal fees and nonfinancial support from Sanofi, personal fees from Teva; Christine Lebrun‐Frénay: fees for consulting or lectures from Novartis, Genzyme, Roche; Jérôme De Sèze: consulting and lecturing fees, travel grants and unconditional research support from Biogen, Genzyme, Novartis, Roche, Sanofi Aventis and Teva Pharma; David Axel Laplaud: served on scientific advisory boards for Roche, Sanofi, Novartis, MedDay, Merck and Biogen, received conference travel support and/or speaker honoraria from Novartis, Biogen, Roche, Sanofi, Celgene and Merck and received research support from Fondation ARSEP and Agence Nationale de la Recherche; Olivier Gout: nothing to disclose; Aurélie Ruet: consultancy fees, speaker fees, research grants (non‐personal), or honoraria approved by the institutions from Novartis, Biogen Idec, Genzyme, Medday, Roche, Teva and Merck; Thibaud Moreau: fees as scientific adviser from Biogen, Medday, Novartis, Genzyme, Sanofi; Olivier Casez: funding for travel and honoraria from Biogen, Merck Serono, Novartis, Sanofi‐Genzyme and Roche; Pierre Clavelou: consulting and lecturing fees, travel grants and unconditional research support from Actelion, Biogen, Genzyme, Novartis, Medday, Merck Serono, Roche, and Teva Pharma; Eric Berger: honoraria and consulting fees from Novartis, Sanofi Aventis, Biogen, Genzyme, Roche and Teva Pharma; Hélène Zephir: consulting or lectures, and invitations for national and international congresses from Biogen, Merck, Teva, Sanofi‐Genzyme, Novartis and Bayer, as well as research support from Teva and Roche, and academic research grants from Académie de Médecine, LFSEP, FHU Imminent and ARSEP Foundation; Guillaume Brocard: nothing to disclose; Romain Casey: nothing to disclose; Christine Lombard: nothing to disclose; Sophie Trouillet‐Assant: nothing to disclose; Eric Thouvenot : consulting and lecturing fees, travel grants or unconditional research support from the following pharmaceutical companies: Actelion, Biogen, Celgene, Genzyme, Merck Serono, Novartis, Roche, Teva pharma.

## Funding information

The study was funded by CHU de Nimes and has also been supported by a grant provided by the French State and handled by the "Agence Nationale de la Recherche," within the framework of the "Investments for the Future" programme, under the reference ANR‐10‐COHO‐002 Observatoire Français de la Sclérose en plaques (OFSEP).

## Supporting information


**Supplementary Figure S1.** Comparison of Simoa^TM^ and Ella^TM^ platforms at low serum NfL levels. A: Repeatability tests of both platforms using samples from one HC and from one RRMS patient tested 30 times. For Simoa^TM^, average NfL concentrations were 6.55 pg/ml and 14.22 pg/ml and CVs were 11.3% and 8.1%, respectively. For Ella^TM^, average serum NfL concentrations were 8.60 pg/ml and 38.38 pg/ml and CVs were 12.8% and 8.9%, respectively, as indicated on the graph. B: Spearman correlation (r) between NfL concentration values obtained by the Ella^TM^ compared to the Simoa^TM^ instruments in a cohort of 29 HCs (r = 0.76, *p* < 0.0001).

**Supplementary Material and Methods.** Origin of serum samples.

## Authors

**Table:**

Name	Location	Role	Contribution
Audrey Gauthier, MSc	École Pratique des Hautes Études, Paris	Author	major role in the acquisition of data analysis or interpretation of the data drafting or revising the manuscript for intellectual content
Sébastien Viel, PharmD, PhD	Hospices Civils de Lyon, Lyon	Author	design or conceptualization of the study analysis or interpretation of the data drafting or revising the manuscript for intellectual content
Magali Perret, MSc	Hospices Civils de Lyon, Lyon	Author	major role in the acquisition of data
Guillaume Brocard, MSc	Hospices Civils de Lyon, Lyon	Author	major role in the acquisition of data
Romain Casey, PhD	Hospices Civils de Lyon, Lyon	Author	design or conceptualization of the study analysis or interpretation of the data drafting or revising the manuscript for intellectual content
Christine Lombard, MSc	Hospices Civils de Lyon, Lyon	Author	major role in the acquisition of data
Sabine Laurent‐Chabalier, PhD	CHU de Nîmes, Nîmes	Author	analysis or interpretation of the data drafting or revising the manuscript for intellectual content
Marc Debouverie, MD, PhD	CHU de Nancy, Nancy	Author	major role in the acquisition of data
Gilles Edan, MD, PhD	CHU Pontchaillou, Rennes	Author	major role in the acquisition of data
Sandra Vukusic, MD, PhD	Hospices Civils de Lyon, Lyon	Author	major role in the acquisition of data
Christine Lebrun‐Frénay, MD, PhD	CHU Pasteur, Nice	Author	major role in the acquisition of data
Jérôme De Sèze, MD, PhD	CHU de Strasbourg, Strasbourg	Author	major role in the acquisition of data
David Axel Laplaud, MD, PhD	CHU de Nantes, Nantes	Author	major role in the acquisition of data
Giovanni Castelnovo, MD	CHU de Nîmes, Nîmes	Author	major role in the acquisition of data
Olivier Gout, MD	Fondation Rotschild, Paris	Author	major role in the acquisition of data
Aurélie Ruet, MD, PhD	CHU de Bordeaux, Bordeaux	Author	major role in the acquisition of data
Thibault Moreau, MD, PhD	CHU de Dijon, Dijon	Author	major role in the acquisition of data
Olivier Casez, MD	CHU de Grenoble, Grenoble	Author	major role in the acquisition of data
Pierre Clavelou, MD, PhD	CHU de Clermont‐Ferrand, Clermont‐Ferrand	Author	major role in the acquisition of data
Eric Berger, MD	CHU de Besançon, Besançon	Author	major role in the acquisition of data
Hélène Zephir, MD, PhD	CHU de Lille, Lille	Author	major role in the acquisition of data
Sophie Trouillet‐Assant, PhD	Hospices Civils de Lyon, Lyon	Author	design or conceptualization of the study analysis or interpretation of the data drafting or revising the manuscript for intellectual content
Eric Thouvenot, MD, PhD	CHU de Nîmes, Nîmes	Author	major role in the acquisition of data design or conceptualization of the study analysis or interpretation of the data drafting or revising the manuscript for intellectual content

### Co‐investigators

*List of OFSEP investigators

(Steering Committee, Principal investigators, Biology group)

### Steering Comittee

Romain Casey, PhD, Observatoire français de la sclérose en plaques (OFSEP), Centre de coordination national, Lyon/Bron, France;

François Cotton, MD, Hospices civils de Lyon, Hôpital Lyon sud, Service d’imagerie médicale et interventionnelle, Lyon/Pierre‐Bénite, France;

Jérôme De Sèze, MD, Hôpitaux universitaire de Strasbourg, Hôpital de Hautepierre, Service des maladies inflammatoires du système nerveux – neurologie, Strasbourg, France;

Pascal Douek, MD, Union pour la lutte contre la sclérose en plaques (UNISEP), Ivry‐sur‐Seine, France;

Francis Guillemin, MD, CIC 1433 Epidémiologie Clinique, Centre hospitalier régional universitaire de Nancy, Inserm et Université de Lorraine, Nancy, France;

David Laplaud, MD, Centre hospitalier universitaire de Nantes, Hôpital nord Laennec, Service de neurologie, Nantes/Saint‐Herblain, France;

Christine Lebrun‐Frenay, MD, Centre hospitalier universitaire de Nice, Université Nice Côte d’Azur, Hôpital Pasteur2, Service de neurologie, Nice, France;

Lucilla Mansuy, Hospices civils de Lyon, Département de la recherche clinique et de l’innovation, Lyon, France;

Thibault Moreau, MD, Centre hospitalier universitaire Dijon Bourgogne, Hôpital François Mitterrand, Service de neurologie, maladies inflammatoires du système nerveux et neurologie générale, Dijon, France;

Javier Olaiz, PhD, Université Claude Bernard Lyon 1, Lyon ingéniérie projets, Lyon, France;

Jean Pelletier, MD, Assistance publique des hôpitaux de Marseille, Centre hospitalier de la Timone, Service de neurologie et unité neuro‐vasculaire, Marseille, France;

Claire Rigaud‐Bully, Fondation Eugène Devic EDMUS contre la sclérose en plaques, Lyon, France;

Bruno Stankoff, MD, Assistance publique des hôpitaux de Paris, Hôpital Saint‐Antoine, Service de neurologie, Paris, France;

Sandra Vukusic, MD, Hospices civils de Lyon, Hôpital Pierre Wertheimer, Service de neurologie A, Lyon/Bron, France;

Hélène Zephir, MD, Centre hospitalier universitaire de Lille, Hôpital Salengro, Service de neurologie, Lille, France;

### Investigators

Marc Debouverie, MD, Centre hospitalier régional universitaire de Nancy, Hôpital central, Service de neurologie, Nancy, France;

Gilles Edan, MD, Centre hospitalier universitaire de Rennes, Hôpital Pontchaillou, Service de neurologie, Rennes, France;

Romain Marignier, MD, Hospices civils de Lyon, Hôpital Pierre Wertheimer, Service de neurologie A, Lyon/Bron, France;

Nicolas Collongues, MD, Hôpitaux universitaire de Strasbourg, Hôpital de Hautepierre, Service des maladies inflammatoires du système nerveux – neurologie, Strasbourg, France;

Mikaël Cohen, MD, Centre hospitalier universitaire de Nice, Université Nice Côte d’Azur, Hôpital Pasteur, Service de neurologie, Nice, France;

Olivier Gout, MD, Fondation Adolphe de Rothschild de l’œil et du cerveau, Service de neurologie, Paris, France;

Sandrine Wiertlewsky, MD, Centre hospitalier universitaire de Nantes, Hôpital nord Laennec, Service de neurologie, Nantes/Saint‐Herblain, France;

Eric Thouvenot, MD, Centre hospitalier universitaire de Nîmes, Hôpital Carémeau, Service de neurologie, Nîmes, France;

Pierre Clavelou, MD, Centre hospitalier universitaire de Clermont‐Ferrand, Hôpital Gabriel‐Montpied, Service de neurologie, Clermont‐Ferrand, France;

Jonathan Ciron, MD, Centre hospitalier universitaire de Toulouse, Hôpital Purpan, Service de neurologie inflammatoire et neuro‐oncologie, Toulouse, France;

Eric Berger, MD, Centre hospitalier régional universitaire de Besançon, Hôpital Jean Minjoz, Service de neurologie, Besançon, France;

Aurélie Ruet, MD, Centre hospitalier universitaire de Bordeaux, Hôpital Pellegrin, Service de neurologie, Bordeaux, France;

Agnès Fromont, MD, Centre hospitalier universitaire Dijon Bourgogne, Hôpital François Mitterrand, Service de neurologie, maladies inflammatoires du système nerveux et neurologie générale, Dijon, France;

Olivier Casez, MD, Centre hospitalier universitaire Grenoble‐Alpes, Site nord, Service de neurologie, Grenoble/La Tronche, France;

Pierre Labauge, MD, Centre hospitalier universitaire de Montpellier, Hôpital Gui de Chauliac, Service de neurologie, Montpellier, France;

Abir Wahab, MD, Assistance publique des hôpitaux de Paris, Hôpital Henri Mondor, Service de neurologie, Créteil, France;

Gilles Defer, MD, Centre hospitalier universitaire de Caen Normandie, Service de neurologie, Hôpital Côte de Nacre, Caen, France;

Philippe Cabre, MD, Centre hospitalier universitaire de Martinique, Hôpital Pierre Zobda‐Quitman, Service de Neurologie, Fort‐de‐France, France;

Nicolas Maubeuge, MD, Centre hospitalier universitaire de Poitiers, Site de la Milétrie, Service de neurologie, Poitiers, France;

Claire Giannesini, MD, Assistance publique des hôpitaux de Paris, Hôpital Saint‐Antoine, Service de neurologie, Paris, France;

Aude Maurousset, MD, Centre hospitalier régional universitaire de Tours, Hôpital Bretonneau, Service de neurologie, Tours, France;

Hélène Zephir, MD, Centre hospitalier universitaire de Lille, Hôpital Salengro, Service de neurologie, Lille, France;

Alexis Montcuquet, MD, Centre hospitalier universitaire Limoges, Hôpital Dupuytren, Service de neurologie, Limoges, France;

Olivier Heinzlef, MD, Centre hospitalier intercommunal de Poissy Saint‐Germain‐en‐Laye, Service de neurologie, Poissy, France;

Elisabeth Maillart, MD, Assistance publique des hôpitaux de Paris, Hôpital de la Pitié‐Salpêtrière, Service de neurologie, Paris, France;

Bertrand Audoin, MD, Assistance publique des hôpitaux de Marseille, Centre hospitalier de la Timone, Service de neurologie et unité neuro‐vasculaire, Marseille, France;

Abdullatif Al‐Khedr, MD, Centre hospitalier universitaire d’Amiens Picardie, Site sud, Service de neurologie, Amiens, France;

## Biology group

David Laplaud, Centre hospitalier universitaire de Nantes, Hôpital nord Laennec, Service de neurologie, Nantes/Saint‐Herblain, France;

Romain Marignier, MD, Hospices civils de Lyon, Hôpital Pierre Wertheimer, Service de neurologie A, Lyon/Bron, France;

Eric Thouvenot, Centre hospitalier universitaire de Nîmes, Hôpital Carémeau, Service de neurologie, Nîmes, France;

Guillaume Brocard, Observatoire français de la sclérose en plaques (OFSEP), Centre de coordination national, Lyon/Bron, France;

Romain Casey, Observatoire français de la sclérose en plaques (OFSEP), Centre de coordination national, Lyon/Bron, France;

Nathalie Dufay, NeuroBioTec, Hôpital Neurologique Pierre Wertheimer, Hospices Civils de Lyon, Lyon/Bron, France;

Caroline Barau, Laboratoire de la PRB, Centre d’Investigation Clinique (CIC), Groupe Hospitalier Henri Mondor, Créteil, France;

Shaliha Bechoua, Etablissement Français du Sang, Service Biothèque‐CRB, Dijon, France;

Gilda Belrose, Centre de Ressources Biologiques de la Martinique (CeRBiM), CHU de Martinique Pierre ZOBDA‐QUITMAN, Fort‐de‐France, France;

Juliette Berger, CRB Auvergne ‐ CHU Estaing, Clermont‐Ferrand, France;

Marie‐Pierrette Chenard, CRB, UF 6337, Département de Pathologie, Hôpital de Hautepierre, Hôpitaux Universitaires de Strasbourg, Strasbourg, France;

Mireille Desille‐Dugast, CRB, Laboratoire de Cytogénétique et Biologie Cellulaire, CHU Pontchaillou, Rennes, France;

Esther Dos Santos, Service de Biologie médicale, Poissy, France;

Arianna Fiorentino, CRB HUEP‐SU, Faculté de médecine site Saint Antoine, Paris, France;

Sylvie Forlani, Banque ADN & Cellules‐ICM U1127, PRB, GH Pitié‐Salpêtrière, Paris, France;

Géraldine Gallot, CRB, UF 7296, CHU de Nantes, Hôtel Dieu, Institut de biologie, Nantes, France;

Patrick Gelé, CRB/CIC1403, Centre de Biologie Pathologie Génétique, Lille, France;

Michèle Grosdenier, EFS, CHU de Poitiers, Poitiers, France;

Yves‐Edouard Herpe, Biobanque de Picardie ‐ CHU Amiens‐Picardie, Amiens, France;

Julien Jeanpetit, Centre de Ressources Biologiques Plurithématique (CRB‐P), Bordeaux Biothèques Santé (BBS), Pôle de Biologie et de Pathologie, CHU de Bordeaux, Bordeaux, France;

Caroline Laheurte, Etablissement Français du Sang, Besançon, France;

Hélène Legros, CHU Caen Normandie, Caen, France;

Sylvain Lehmann, CHU Saint Eloi, IRMB, Biochimie Protéomique Clinique, Montpellier, France;

Céline Loiseau, CRB, Laboratoire de cytogénétique, CHU de Nîmes, Nîmes, France;

Sandra Lomazzi, CRB Lorrain‐ CHRU Nancy, Vandoeuvre‐les‐Nancy, France;

Philippe Lorimier, Centre de Ressources Biologiques, Institut de Biologie et de Pathologie, CHU Albert Michallon, Grenoble, France;

Mikael Mazighi, Fondation Ophtalmologique Adolphe de Rothschild, Centre de Ressources Biologiques, Paris, France;

Samantha Montagne, CHRU Bretonneau, CRB, EFS, Tours, France.

Bénédicte Razat, CRB Toulouse Bio Ressources, Toulouse, France;

Noémie Saut, Service d'Hématologie Biologique, CHU Timone adultes, Marseille, France;

Emilie Villeger, CRBIoLim, CHU Dupuytren, Limoges, France;

Kevin Washetine, CHU de Nice, Hôpital Pasteur 1, Nice, France.
