# Supplementary figures and images for: Comparison of SimoaTM and EllaTM to assess serum neurofilament‐light chain in multiple sclerosis
Source: Ann Clin Transl Neurol. 2021 Apr 8;8(5):1141–50. doi: 10.1002/acn3.51355 (PMC8108418; doi:10.1002/acn3.51355)

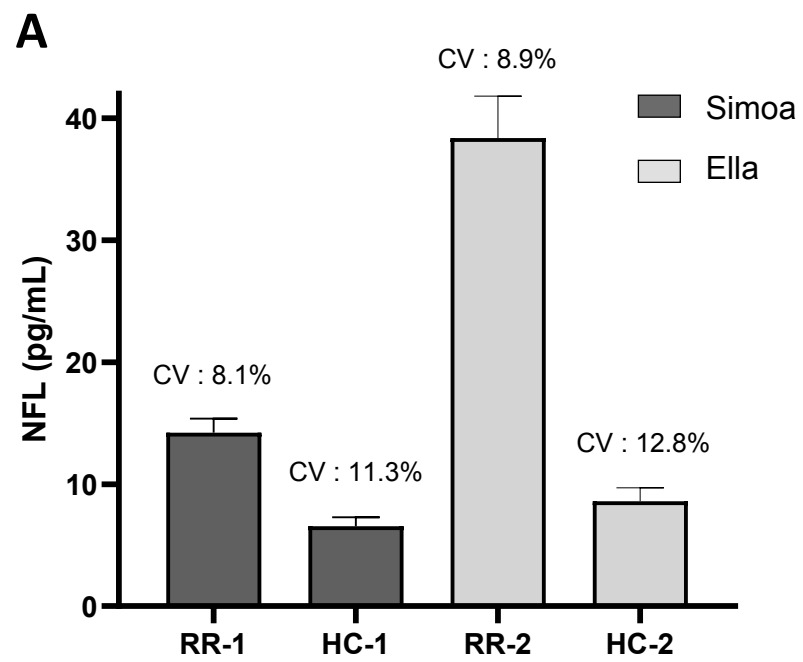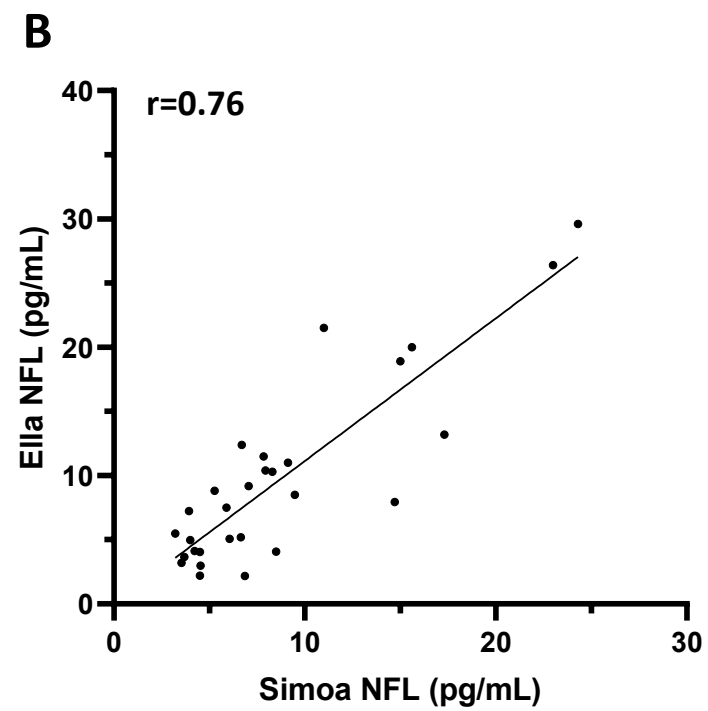

Supplement: Supplementary file 1 — Supplementary Figure S1. Comparison of SimoaTM and EllaTM platforms at low serum NfL levels. A: Repeatability tests of both platforms using samples from one HC and from one RRMS patient tested 30 times. For SimoaTM, average NfL concentrations were 6.55 pg/ml and 14.22 pg/ml and CVs were 11.3% and 8.1%, respectively. For EllaTM, average serum NfL concentrations were 8.60 pg/ml and 38.38 pg/ml and CVs were 12.8% and 8.9%, respectively, as indicated on the graph. B: Spearman correlation (r) between NfL concentration values obtained by the EllaTM compared to the SimoaTM instruments in a cohort of 29 HCs (r = 0.76, p < 0.0001). [file ACN3-8-1141-s002.pdf]
