# Supplementary material for: Comparison of SimoaTM and EllaTM to assess serum neurofilament‐light chain in multiple sclerosis
Source: Ann Clin Transl Neurol. 2021 Apr 8;8(5):1141–50. doi: 10.1002/acn3.51355 (PMC8108418; doi:10.1002/acn3.51355)
Supplement: Supplementary file 2 — Supplementary Material and Methods. Origin of serum samples. [file ACN3-8-1141-s001.docx]

**Supplementary materials and methods. Origin of serum samples.**

Samples selected from 13 recruiting centers were equally distributed between three 72-well plates. Samples from one healthy control (HC) and one MS patient with active relapsing-remitting MS (RRMS) (during an acute spinal cord relapse) were used as internal controls, and another HC and another RRMS patient were used for repeatability. MS patients were classified as RRMS, progressive (PMS) MS, and whether they presented a relapse (relapse) or disease activity (active = relapse or active MRI lesion within 3 months before sampling) within 3 months before sampling. A group of 29 age-matched HCs was used as control (mean age 42.1 y-o in HCs compared to 40.1 y-o in RRMS, p=0.38, Wilcoxon-Mann-Whitney). According to EFS, the French national blood service, standardized procedures for blood donation and to provisions of the articles R.1243–49 and following ones of the French public health code, a written non-opposition to the use of donated blood for research purposes was obtained from HV. The blood donors’ personal data were anonymized before transfer to our research laboratory. We obtained the favourable notice of the local ethical committee (Comité de Protection des Personnes Sud-Est II, Bâtiment Pinel, 59 Boulevard Pinel, 69,500 Bron) and the acceptance of the French ministry of research (Ministère de lʼEnseignement supérieur, de la Recherche et de lʼInnovation, DC-2008-64) for handling and conservation of these samples.
